# Involvement of the Innate Immune System in the Pathogenesis of Chronic Obstructive Pulmonary Disease

**DOI:** 10.3390/ijms23020985

**Published:** 2022-01-17

**Authors:** Stanislav Kotlyarov

**Affiliations:** Department of Nursing, Ryazan State Medical University, 390026 Ryazan, Russia; SKMR1@yandex.ru

**Keywords:** COPD, innate immune system, inflammation, bronchial epithelium, macrophages, TLR, bacterial colonization

## Abstract

Chronic obstructive pulmonary disease (COPD) is a common, socially significant disease characterized by progressive airflow limitation due to chronic inflammation in the bronchi. Although the causes of COPD are considered to be known, the pathogenesis of the disease continues to be a relevant topic of study. Mechanisms of the innate immune system are involved in various links in the pathogenesis of COPD, leading to persistence of chronic inflammation in the bronchi, their bacterial colonization and disruption of lung structure and function. Bronchial epithelial cells, neutrophils, macrophages and other cells are involved in the development and progression of the disease, demonstrating multiple compromised immune mechanisms.

## 1. Introduction

Chronic obstructive pulmonary disease (COPD) is a common, socially significant disease [1]. It is one of the leading causes of hospital admissions, disability and mortality [2,3]. The prevalence of the disease shows negative trends in many countries of the world, which is associated with a high prevalence of smoking and exposure to aeropollutants [4]. Moreover, epidemiological data on the prevalence of COPD differ depending on the methods of diagnosis and classification of the disease used [5].

COPD is associated with a significant economic burden on patients and their families, and may also have an impact on the health care system in some countries [6]. This effect is due to the negative impact on work capacity and prognosis, which are related both to the disease itself and to comorbid pathology, which is widespread in patients with COPD. Indeed, cardiovascular disease, osteoporosis and cachexia are often associated with COPD and contribute to the overall clinical picture.

COPD is a chronic disease, the leading cause of which is associated with the long-term inhalation of components of tobacco smoke [7]. Chronic inflammation persists for many years, and the subsequent remodeling of the bronchi leads to the development of airway obstruction and increased tissue hypoxia. Hypoxia and systemic inflammation contribute significantly to the development of pulmonary and extrapulmonary clinical heterogeneity of COPD, which influences the natural history of the disease [8,9].

Spirometry is considered to be a key method for diagnosing COPD and should be used more widely in clinical practice [10,11]. Spirometry screening improves diagnosis, given that patients may go a long time without seeking medical care, considering respiratory symptoms as a natural manifestation of smoking. In this regard, early diagnosis and cessation of smoking improve the therapeutic effectiveness of patient management. The diagnosis of COPD should be assumed in long-term smokers over 40 years of age who have chronic respiratory symptoms, such as cough with sputum production and shortness of breath [11]. Assessment of respiratory symptoms is an important clinical tool to assess the functional status of COPD patients [12].

Infectious exacerbations of COPD are associated with a worsening of the clinical manifestations of the disease, especially with a high frequency of exacerbations [13]. This variant course of COPD is of great clinical interest because the frequency of exacerbations influences the clinical picture and prognosis. Bacterial colonization is an important factor of COPD progression [14]. The normal bronchi are known to be inhabited by a diverse microbial community, which contributes to the regulation of the immune response in the lungs. Increased bacterial load and changes in the type of colonizing bacteria in COPD are associated with increased inflammation in the airways and a more rapid decline in forced expiratory volume in the first second (FEV_1_) [15,16].

There is increasing evidence that dysregulation of the innate immune system is associated with the development and progression of COPD. Research in recent years has significantly expanded our understanding of the mechanisms available to the innate immune system to protect the organism. Inflammation in the bronchi involves many cells, which demonstrate complex cross-linkages in the innate immune response. The role of macrophages in inflammation in COPD has been the subject of numerous studies that have expanded the understanding of their involvement in immune mechanisms.

The aim of this review is to discuss the role of the innate immune system in the development of COPD and how impaired innate immune system mechanisms are associated with the clinical heterogeneity of COPD.

## 2. The Innate Immune System

The innate immune system is an evolutionarily ancient defense system that allows the organism to maintain constancy of its macromolecular composition by detecting and removing foreign molecules and providing resistance to infectious agents [17]. Given that large volumes of inhaled air containing various aeropollutants and microorganisms pass through the lungs on a daily basis, this organ requires serious immune protection.

The innate immune system uses many cellular and humoral mechanisms [18]. The innate immune system is thought to react nonspecifically to different agents. The consequence of this can be damage to one’s own cells. In addition, micro-organisms have developed a wide range of strategies that are used to colonize the bronchi [19]. Recent evidence suggests that some mechanisms of the innate immune system allow the formation of immunological memory [20], highlighting the complexity and underestimation of this part of the immune system.

Cigarette smoke contains a significant number of different components, including free radicals and reactive oxygen species involved in lipid peroxidation [21]. Exposure to components of cigarette smoke can affect immune processes, leading not only to impaired infectious lung protection, but also to chronic inflammation and related resulting effects on tissue structure and function.

Thus, the role of the innate immune system in chronic diseases such as COPD and atherosclerosis is the focus of increased attention.

### 2.1. The Innate Immune Function of Airway Epithelial Cells

Respiratory epithelial cells are exposed to many different inhaled particles and gases every day. These cells form a barrier between the external and internal environment of the body and play a key role in organizing inflammatory and immune responses in the lungs [22]. The accumulated knowledge has expanded the understanding of the role of respiratory epithelium in the innate immune system of the lungs [23]. The function of the epithelium in protecting the host from infection involves several known mechanisms.

The first line of defense is provided by the barrier function of epithelial cells through dense intercellular junctions and the mechanical clearance of the airways, which is considered a key protective mechanism of the epithelium [24]. Epithelial mucus production and mucociliary clearance are mechanisms that ensure bronchial clearance of inhaled and aspirated particles and pathogens. This function is provided by the production of mucus as well as by the presence of coordinated beating epithelial cell cilia.

Detailed analysis has shown that mucus is not homogeneous in composition and function, but consists of two layers that together form the airway surface liquid [25]. The first layer is the layer of periciliary liquid. The periciliary liquid has a low viscosity which enables the cilia to beat rapidly (about 8–15 Hz) [25]. This layer is important for normal mucociliary clearance, as the co-ordinated beating of the cilia ensures the transport of the overlying mucus layer where inhaled substances and pathogens are retained [26]. The second layer is represented by epithelial mucus, which consists mainly of water, but also mucins, proteins, lipids, salts and other molecules. Epithelial mucus acts as a physical barrier for many pathogens [27,28]. Mucins, through their side chains, are able to bind to a variety of particles that reach the epithelium and can remove them from the respiratory tract through the mechanism of mucociliary clearance [27,29,30].

In COPD, these mechanisms may be impaired, accompanied by mucus hypersecretion. Mucus hypersecretion is associated with mucins, of which MUC5AC is considered the most important [31,32]. MUC5AC is produced predominantly by goblet cells of the bronchial epithelium [33]. Overproduction of MUC5AC in COPD leads to increased airway obstruction by mucus [31]. Elevated concentrations of MUC5AC in the airways may contribute to the development and progression of COPD and are associated with clinical manifestations of the disease, including declines FEV_1_ and increased risk of exacerbations [34]. Increased sputum production is thought to be one of the clinical characteristics of the bronchial phenotype of COPD. In addition to affecting the quantitative and qualitative parameters of mucus, exposure to cigarette smoke reduces cilia length, which also reduces the efficiency of mucociliary clearance [35].

In addition to the first line of defense of the epithelium, which is provided by the mechanical cleansing of the respiratory tract, another important mechanism is considered to be chemical and immune defense through the production of certain molecules [25]. These substances belong to different chemical groups and are involved in the regulation of the immune response.

Antimicrobial peptides such as defensins and cathelicidins play an important role in the protective function that bronchial epithelial cells provide [26,36,37]. Defensins have antimicrobial activity, affecting membrane permeability in bacteria and fungi [38,39,40]. They also exhibit a number of antiviral effects, including a direct effect on viral envelopes, capsids and glycoproteins. They have also been found to inhibit the entry of some viruses into cells and their ability to prevent viral replication [41]. These findings are consistent with the fact that defensin expression in the respiratory epithelium is increased by exposure to pathogenic bacteria or viruses [26,42,43].

Elevated levels of β-defensin-1 protein may be considered a marker of COPD [44]. Sputum beta-defensin-1 has a negative correlation with FEV_1_ [45]. Furthermore, primary human bronchial epithelial cells in patients with COPD produce higher levels of beta-defensin-1 than epithelial cells from healthy donors when infected with nontypeable *Haemophilus influenza (NTHi)* in an in vitro experiment [45]. Expression of human beta-defensin-2 (hBD-2) was shown to be elevated in the distal airways of patients with COPD [46]. Moreover, hBD-2 mRNA levels correlated positively with interleukin-8 (IL-8) mRNA levels, and correlated negatively with predicted values of FEV_1_ and FEV_1_/FVC (forced vital capacity) [46]. In another study, smoking was associated with a significant reduction in hBD-2 levels in sputum in patients with acute *Pseudomonas aeruginosa* [37]. Moreover, the release of the proinflammatory cytokine IL-8 from epithelial cells is increased when smoke exposure and bacterial infection are combined. These findings confirm the negative effects of smoking on the protective function of the epithelial barrier.

Another representative of antimicrobial peptides in humans is LL-37, which belongs to the cathelicidin class [26]. LL-37 is induced by inflammatory or infectious stimuli and exhibits antimicrobial activity against Gram-positive and Gram-negative bacteria [47,48]. It also binds and neutralizes lipopolysaccharide (LPS) activity, protecting against endotoxic shock [49]. LL-37 also shows activity against fungi and viruses, can affect apoptosis, and has chemotactic activity for neutrophils, monocytes, mast cells and T cells [50,51,52]. In addition to its antimicrobial properties, LL-37 may be involved in tissue repair and wound healing [53]. LL-37 production is also induced by short-chain fatty acids, such as butyrate, which are produced by gut bacteria, demonstrating the lung-gut axis [54,55]. LL-37 exerts its action via several receptors such as formyl peptide receptor 2 (FPR2), epidermal growth factor receptor (EGFR), erb-b2 receptor tyrosine kinase 2 (ErbB2) and purinergic receptor P2X7 (P2X7R) [53,56]. In addition, this peptide can exert its antimicrobial action directly through electrostatic interaction with negatively charged bacterial membranes [53].

Studies suggest that LL-37 may be involved in the pathogenesis of COPD [56]. The increased expression of LL-37 in the airways and lung alveoli in COPD has been found. Because LL-37 can promote IL-8 production and induce apoptosis in bronchial and alveolar epithelial cells, increased levels of LL-37 in the sputum of patients with COPD have been associated with airway obstruction and a worsening of the clinical course of COPD [50]. Another mechanism that is associated with a negative effect on clinical presentation is that LL-37 enhances MUC5AC mucin production in the airways of COPD via the TACE-TGF-α-EGFR pathway [57]. LL-37 levels in sputum have been shown to be higher during exacerbations of COPD induced by *NTHi* and *Moraxella catarrhalis* compared to baseline [58]. Interestingly, plasma LL-37 levels were lower in COPD patients at high risk of frequent exacerbations than in normal patients [59]. These data suggest that decreased plasma LL-37 levels may increase the risk of exacerbations in patients with COPD. In this regard, it should be noted that the cathelicidin LL-37 also plays a protective role by affecting barrier function and tight junction proteins in the bronchial epithelium against exposure to cigarette smoke [60].

In addition to antimicrobial peptides, airway epithelial cells are involved in the production of bioactive lipid mediators of inflammation and inflammation resolution [61]. Eicosanoids are synthesized from some polyunsaturated fatty acids, mainly arachidonic acid, which can be released from membrane phospholipids by phospholipase A2. Secretory phospholipase A2 (sPLA2) is secreted from ciliated cells and acts on the goblet cells to induce production of MUC5AC, Leukotriene B4 (LTB4) and Leukotriene C4 (LTC4) [62]. Goblet cells are considered to be effector pro-inflammatory cells in the airways [62].

Arachidonic acid can be converted into eicosanoid mediators by three enzymatic pathways: cyclooxygenase (COX), lipoxygenase (LO) and cytochrome P450 (CYP) [63].

In an experiment on Human Bronchial Epithelial Cells (HBEC) line, the production of mainly prostaglandins (PGE2, PGD2) and epoxides (e.g., 14,15-EET (14,15-epoxy-5Z,8Z,11Z-eicosatrienoic acid)) has been shown. However, exposure of cultured cells to IL-13 and IL-4 resulted in a significant increase in 15-Hydroxyeicosatetraenoic acid (15-HETE) production and a moderate increase in 12-HETE concentration and a decrease in PG production [64]. 15-HETE is considered an important participant in inflammation due to its ability to inhibit the migration of inflammatory cells in response to chemotactic factors [64,65]. In addition, 15-HETE is involved in lipoxin synthesis and can modulate cell proliferation [66,67,68].

PGE2 is an eicosanoid derived from arachidonic acid by COX-2. It induces many different effects by acting through four different G-protein coupled receptors (EP1-4) [69]. PGE2 is known as a respiratory smooth muscle relaxant and may also have bronchoprotective properties, promoting cell growth in the bronchial epithelium [62,70,71,72]. PGE2 also has anti-inflammatory properties via the activation of the EP4 receptor [73].

PGE2 reduces the production of several pro-inflammatory cytokines, such as IL-8, IL-12, monocyte chemotactic protein (MCP)-1 and granulocyte-macrophage colony-stimulating factor (GM-CSF), which are involved in leukocyte migration [74,75,76,77].

Some bacteria use PGE2 to counteract inflammation, which contributes to their colonization of the airways of patients with COPD and may play a role in disease progression [78]. In particular, *Streptococcus pneumoniae*, *M. catarrhalis* and *NTHi* have been shown to induce COX-2 expression and prostaglandin E2 (PGE2) production in the respiratory epithelium [78,79,80]. It was also shown that in addition to *M. catarrhalis* induced COX-2 expression and increased PGE2 production in lung epithelial cells, EP2 and EP4 receptors were also activated in these cells [78]. Thus, the increased concentration of prostaglandin PGE2 in the lungs of patients may be related to the pathogenesis of COPD [81]. This seems important given the important immunomodulatory function of PGE2 on dendritic cells [82].

Nitric oxide (NO) is another biologically active substance that is involved in many physiological and immune processes in the lungs [83]. The role of nitric oxide is well known from the field of vascular biology, where its bioavailability is related to endothelial dysfunction [84]. Most of the exhaled NO is due to constitutive expression of NOS2 [85,86].

In the airway epithelium, NO is involved in the mucociliary function, where it upregulates ciliary beat frequency [87]. NO has also been shown to be involved in the regulation of epithelial ion transport [88] and may also contribute to the restoration of epithelial integrity [89]. In addition, NO is considered to be a universal player in the immune system [90]. NO modulates inflammation by regulating the production of epithelial inflammatory mediators and also directly contributes to innate immune defense [90]. Levels of NO can both increase and inhibit NF-kB activation [83]. Immune cells use NO to destroy pathogens, which is an evolutionarily ancient mechanism [91]. NO acts in a non-specific manner, killing various targets, including bacteria, protozoa and viruses [92]. However, due to lack of specificity, this mechanism may also have negative potential and, if not adequately regulated, may lead to concomitant damage to normal cells and tissues [86,93].

Pro-inflammatory cytokines, which are released by mononuclear cells, can stimulate the expression of iNOS [94,95]. NO production by epithelial cells is enhanced in the presence of LPS, interferon gamma, IL-1β, TNF-α [96].

Patients with COPD had significantly increased iNOS mRNA and protein levels compared to non-smokers and smokers with normal lung function. In addition, there was a negative correlation between iNOS protein levels and lung function parameters such as FEV_1_ and FEV_1_/FVC [97].

Airway epithelial cells can be an important source of inflammatory mediators in smoking and COPD, such as IL-1β, IL-8, TNF-α and GM-CSF [98,99]. IL-8 is an important mediator of airway inflammation and innate immunity. It is a chemoattractant for neutrophil cells [100]. IL-8 levels are elevated in the induced sputum of patients with COPD [100,101].

Cigarette smoke can increase IL-8 production by bronchial epithelial cells, acting in a concentration- and time-dependent manner. The concentration of IL-8 was higher in proximal airway samples than in distal alveolar samples [98]. The combination of smoke exposure and bacterial infection also increased the release of the proinflammatory cytokine IL-8 from epithelial cells [37]. The cytochrome P450-derived signaling molecules 11,12-EET and 14,15-EET caused a decrease in IL-8 production induced by smoking. Reduced levels of EET may decrease the anti-inflammatory capacity of epithelial cells, representing another mechanism of inflammation regulation [100].

Thus, the bronchial epithelium demonstrates the presence of various tools for initiating and maintaining inflammation, and also has some anti-inflammatory mechanisms. This function of bronchial epithelial cells is related to their ability to detect pathogens and induce a response, through the expression of pattern recognition receptors. The expression and function of TLRs on airway epithelial cells is of increasing interest. Toll-like receptors are evolutionarily ancient pattern recognition receptors of the innate immune system [102]. They recognize pathogen-associated molecular patterns (PAMPs) and trigger innate immune responses involved in the first line of host defense against microbial infection, including cytokine production.

TLR1-6 and TLR9 have been shown to be expressed in human airway epithelial cells, and TLR2 and TLR4 are expressed in alveolar epithelial cells [103,104,105]. TLR2 is involved in the detection of Gram-positive bacteria, whereas TLR4 recognizes LPS of Gram-negative bacteria [106].

TLR4 expression is increased in patients with mild to moderate COPD compared to normal controls, but TLR4 expression decreased with increasing disease severity and declining FEV_1_ [107]. Reduced TLR4 expression in the lungs was associated with airflow limitation and emphysema in smokers [108]. In contrast, in another study, TLR4 expression in the bronchial epithelium was increased in severe and very severe stable COPD compared with non-smokers [109]. In addition, NOD1 expression was elevated in the bronchial epithelium in severe and very severe stable COPD compared to patients with mild and moderate stable COPD. In this study, the degree of airway obstruction was positively correlated with increased *P. aeruginosa* bacterial load and decreased *H. influenzae* load in bronchial biopsy samples from smokers and patients with stable COPD [109]. These findings are consistent with the role of TLR4 in removing *H. influenzae* and altering the lung microbiological landscape in severe COPD [109]. Differences in TLR4 expression in the bronchial epithelium in different individuals may reflect the morphological and clinical and functional heterogeneity of COPD, including the development of emphysema and exacerbations. It is known that despite a common etiological factor, the clinical course of COPD, including the development of emphysema, the frequency of exacerbations and the rate of progression of airway obstruction may differ between individuals. TLR2 signalling is also crucial for airway mucin expression in *Mycoplasma pneumoniae* respiratory infection [110].

A crucial feature of the immune function of the bronchial epithelium is the ability to distinguish between harmful pathogens and harmless members of the commensal flora [111]. Gram-positive bacteria have been found to be less able to stimulate cells of the bronchial epithelium, whereas Gram-negative bacteria are readily recognized [103]. These observations correlated with low TLR2 expression and lack of CD36 receptor expression [103]. The above data may indicate that TLR expression levels and coreceptor expression in epithelial cells are a mechanism for regulating the sensitivity of microbial recognition [103,112]. This is important, given that the bronchi are not sterile, but have an extensive microbial landscape that is involved in the immunological tone of the lungs.

Impaired epithelial repair in COPD can lead to a hypercoagulable state, as well as intra-alveolar fibrin accumulation and basal membrane destruction [113]. The presence of alveolar fibrin caused by inhibition of fibrinolysis, in turn, contributes to lung dysfunction and an acute inflammatory response [114].

**Figure 1:**
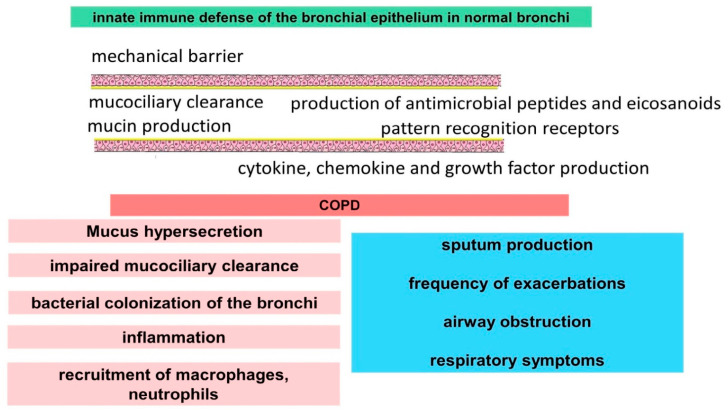
Disruption of innate immune mechanisms of airway epithelial cells in COPD.

Thus, bronchial epithelial cells have a variety of mechanisms to maintain immunological homeostasis and provide microbial protection (Figure 1). These tools can be impaired by smoking, leading to activation and maintenance of inflammation and changes in the composition of bronchial microflora, which is associated with exacerbations. Factors associated with impaired bronchial epithelial function are implicated in the pathogenesis of COPD, demonstrating involvement in increased sputum production and airway obstruction.

### 2.2. Role of Macrophages in the Innate Immune System and Pathogenesis of COPD

Alveolar macrophages, given their open position in the lumen of the alveoli, are in constant contact with inhaled air and thus with various substances and gases. These cells are considered to be key players in the innate immune defense of the lungs [115]. Macrophages control innate immunity by coordinating inflammatory responses as well as directly phagocytizing pathogens.

The pulmonary macrophage system is characterized by heterogeneity in origin, localization and function. Three main populations of this cell type are known, such as alveolar macrophages, interstitial macrophages and cells differentiated from blood monocytes [116]. The macrophage population in the lungs is maintained both by self-renewal of alveolar macrophages and by differentiation of monocytes recruited from the bloodstream [117].

**Figure 2:**
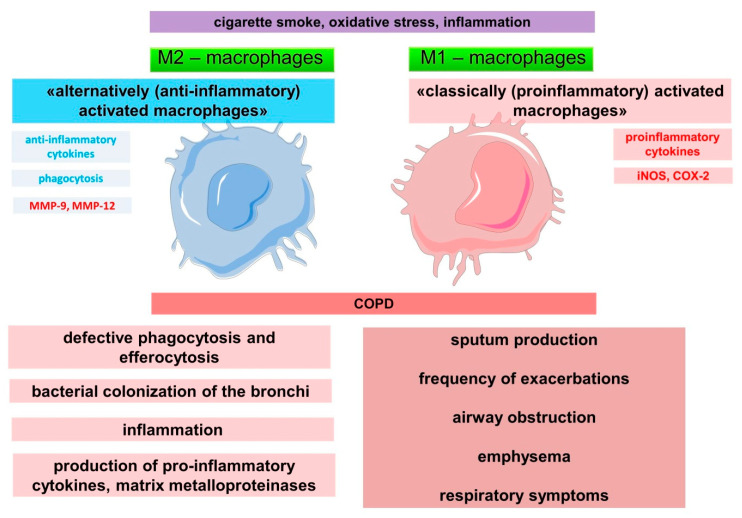
Disruption of innate immune mechanisms of macrophages in COPD.

The ability of alveolar macrophages to self-renew and to maintain an optimal number of macrophages is impaired in COPD. Macrophages are actively involved in inflammation in COPD (Figure 2) [118], as evidenced by data on an increase in their numbers in lung tissue, as well as in sputum and bronchoalveolar lavage fluid [115,119]. Cigarette smoking was associated with a fivefold increase in the number of macrophages in bronchoalveolar lavage [118]. The number of macrophages in the airways correlates with the severity of inflammation, the degree of airflow limitation, and thus with the severity of COPD [118,120]. In addition, macrophages are associated with destruction of the alveolar wall in the development of emphysema. Patients with emphysema have been shown to have a significant increase in the number of macrophages in the parenchyma and alveolar space compared to smokers with normal lung function [121]. These findings suggest an important role for macrophages in emphysema. Indeed, in an experimental mouse model of COPD, depletion of macrophages in the lungs led to a reduction in cigarette smoke-induced emphysema, as well as protection against changes in lung function [122].

The involvement of macrophages in the inflammatory immune response has been the subject of numerous studies. The alveolar macrophage population is not thought to be homogeneous in its involvement in inflammation. According to the classical model, a «classically activated» type 1 (M1) and an «alternatively activated» type 2 (M2) (subtypes M2a, M2b, M2c), and some other types are distinguished [123,124,125]. M1 macrophages are characterized by pro-inflammatory activity, producing factors such as TNF-α, IL-1β, IL-6 and IL-12 [126,127]. M1 macrophages also highly express the enzyme cyclooxygenase 2 (COX 2), inducible nitric oxide synthase (iNOS or NOS2) [123,128]. In contrast, M2 macrophages are involved in tissue remodeling, including through stimulation of macrophage efferocytic functions and the production of anti-inflammatory factors such as IL-10 and IL-4 [129,130]. In addition, M0 macrophages that have not been exposed to any pro- or anti-inflammatory stimuli have been identified [131].

Macrophage polarization is based on changes in cell metabolism [128]. Polarization is driven by humoral factors and changes the role of macrophages in the inflammatory process. The role of individual macrophage phenotypes in the pathogenesis of COPD is unclear. While alveolar macrophages were mostly unpolarized in normal lungs, M1 and M2 polarization was significantly increased in smoking and COPD. Co-expression of M1 and M2 polarization markers in the same alveolar macrophage was shown [132].

Although the total number of macrophages in the respiratory tract in smoking and COPD is significantly increased, phagocytosis and elimination of microorganisms and apoptotic cells are impaired, indicating defective functional properties of macrophages [133]. Impaired phagocytosis in COPD is considered an important cause of disease progression [133]. The severity of COPD has been shown to correlate with impaired phagocytosis by alveolar macrophages for nontypeable *NTHi* and *M. catarrhalis* [134]. These findings suggest that the inability of alveolar macrophages in COPD to mount an adequate phagocytic response may result in impaired bacterial clearance, which is one of the causes of persistent inflammation [134].

The mechanisms of impaired efferocytosis in patients with COPD are not clear. An accumulation of apoptotic epithelial, endothelial and immune cells in the lungs is noted in patients. In addition, the induction of structural apoptosis of airway cells may be responsible for the development of emphysematous changes.

Macrophages are considered «professional» phagocytes and can regulate the phagocytic activity of epithelial cells [135]. Macrophages express various receptors on the cell surface that are involved in the recognition of molecules or ligands. Phagocytosis is activated when several receptors, such as Fc- and complement receptors, as well as receptors for PAMPs, like TLR, are recognized for antigen recognition, which can make phagocytosis more efficient [136,137]. Macrophages express various Toll-like receptors, with TLR1, TLR2, TLR4, TLR5 and TLR6 being expressed on the cell surface and recognizing LPS, lipopeptides and flagellin, whereas TLR3, TLR7, TLR8 and TLR9 are expressed in intracellular compartments (endosomes) and recognize viral DNA and RNA [138]. Importantly, cigarette smoke can stimulate TLR4, thereby enhancing IL-8 production [139].

In addition to TLRs, Nod-like receptor 3 (NLRP3) inflammasome activation may be involved in the pathogenesis of COPD [140,141]. NLRP3 inflamassome is a molecular protein complex that acts as a platform for the maturation of the pro-inflammatory cytokines IL-1β and IL-18, which may contribute to airway inflammation. Inflamassome NLRP3 can be activated in response to a wide range of stimuli, such as PAMPs from invading pathogens and damage-associated molecular patterns (DAMPs) released from dying cells [142]. NLRP3 and IL-1β mRNA are increased in lung tissue in stable COPD and correlate with airway obstruction, but both caspase-1 and ASC (apoptosis-associated speck-like protein containing a CARD) were largely inactive [143]. It has been suggested that NLRP3 activation of the inflammasome occurs during an infectious exacerbation of COPD [140]. Systemic and local activation of NLRP3 airway inflammation could be considered as a prognostic factor for acute exacerbation of COPD [144]. These findings also suggest the presence of NLRP3 inflammation cross-links, in which different cells, including the bronchial epithelium, macrophages and neutrophils, are involved [145,146,147]. It is shown that neutrophils may play a key role in the activation of NLRP3 inflammasome in alveolar macrophages during respiratory viral infection [148].

Alveolar macrophages in COPD have been shown to develop tolerance to repetitive LPS stimulation, but this only occurs for individual cytokines such as TNFα, CCL5 and IL-10. Interestingly, tolerance is not observed for IL-8 and IL-6. IL-8 is a chemoattractant for neutrophils and has higher levels in the lungs of patients with COPD [149]. IL-8 is produced by different cell types, including epithelial cells, macrophages and smooth muscle cells. Bacterial stimulation may be one reason for the increased production of IL-8 in the lungs of patients with COPD, as IL-8 levels in the lungs and neutrophil counts were higher in COPD patients with bacterial colonization [101,149,150]. Repeated stimulation of TLR2 or TLR4 causes persistent production of IL-8, which may lead to increased neutrophilic inflammation in the lungs [149]. Experimentation over time showed different patterns between TNF-α and IL-8 gene expression following TLR4 activation, with IL-8 showing slower induction and longer duration of expression [149].

Other important factors contributing to the progression of COPD are enzymes such as matrix metalloproteinases (MMPs) and cathepsins produced by macrophages [151,152]. Their increased production can lead to damage to lung tissue. The overexpression of MMP-1, MMP-2, MMP-7, MMP-9 and MMP-12 has been shown to be associated with the development of emphysema in the lungs of smokers [152,153,154].

Thus, macrophages are actively involved in the development and progression of COPD, which is related to their impaired function in providing innate immune protection to the lungs.

### 2.3. Role of Other Cells in the Regulation of the Innate Immune System and the Pathogenesis of COPD

Endothelial cells are thought to be involved in the pathogenesis of emphysema. Indeed, observations suggest impaired vascularization of the interalveolar septa in emphysema due to endothelial cell apoptosis [155]. Endothelial cells also demonstrate a role in the innate immune response by participating in the recognition of PAMP and DAMP [156]. Endothelial cells express TLR4 [157]. Interestingly, TLR4 deficiency resulted in spontaneous emphysema in mice in the absence of a significant increase in inflammatory cell numbers and increased endogenous Nox3 activity in endothelial cells [157]. These data highlight the significant role of TLR4 in the maintenance of lung structural integrity and the development of emphysema.

Neutrophils are actively involved in the pathogenesis of COPD, as evidenced by the increased number of activated neutrophils in sputum and bronchoalveolar lavage fluid [101,158,159]. The increase in neutrophil counts in bronchial and induced sputum corresponds to an increase in the rate of lung function decline and the severity of COPD disease [120,160]. As with macrophages, airway neutrophilia in COPD does not contribute to a reduction in airway colonization and bacterial exacerbations [161,162,163]. The causes and mechanisms of increased neutrophil migration in COPD are subjects of study. Various cells are involved, including the airway epithelium, leading to increased levels of several neutrophil chemoattractants, including LTB4, IL-8, growth-related oncogene-alpha (GROα) and epithelial-neutrophil activating peptide (ENA-78 or CXCL5) [164,165,166].

There is growing interest in phenotypes or subtypes of neutrophil cells that have a wide range of functions in inflammation [162]. These phenotypes involve subpopulations such as the classical pro-inflammatory neutrophil, the pro-angiogenic (reparative) neutrophil, and the anti-inflammatory neutrophil [167]. Consistent with this, neutrophils in the lungs exhibit both pro-inflammatory and anti-inflammatory actions, which are necessary to destroy pathogens and resolve inflammation. They express more than 30 different receptors, including G-protein-coupled receptors, Fc receptors, adhesion receptors, cytokine receptors and pattern recognition receptors [162]. Through the release of serine proteases, such as neutrophil elastase (NE), cathepsin G and proteinase 3 (PR3), neutrophils can cause tissue damage [168]. Substrates of neutrophil proteinases include elastin and collagen, which are major components of the extracellular matrix, and their degradation is the morphological basis of COPD progression [162,169,170]. However, a negative association has been shown between the number of neutrophils and the degree of alveolar destruction in smokers, in contrast to alveolar macrophages, which requires further research [171].

Dendritic cells play an important role in initiating the immune response [172,173]. The airways contain a rich network of dendritic cells adjacent to epithelial cells, where cells capture antigens and recognize danger signals [172,174]. Dendritic cells are at the intersection of innate immune system connections and adaptive pulmonary immunity. Dendritic cells can produce profibrogenic cytokines, contributing to airway remodeling [175]. They initiate and can regulate immune responses to inhaled antigens, including viruses and bacteria [174,175].

Smokers with COPD have a significantly reduced number of mature dendritic cells in the bronchial epithelium compared to healthy individuals, which may affect the regulation of the immune response and coordination of the innate and adaptive lung immune system [172,176,177].

**Figure 3:**
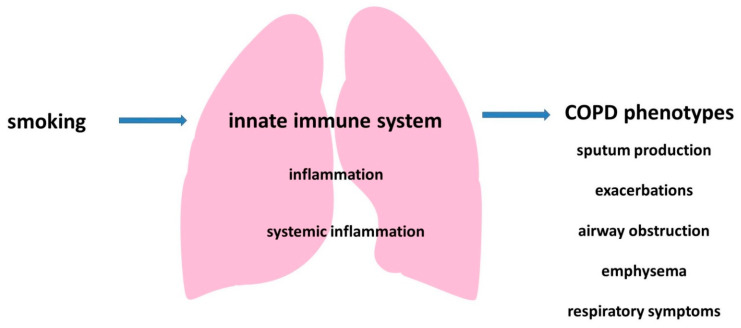
The role of the innate immune system in the development of COPD.

Thus, the pathogenesis of COPD involves different cells of the innate immune system, which leads to the development of local and systemic inflammation and may be associated with some clinical characteristics of the disease (Figure 3).

## 3. Conclusions

COPD, due to chronic inflammation of the airways, is characterized by mucus hypersecretion, bronchial remodeling, and emphysema, resulting in reduced lung function and chronic respiratory symptoms.

The lungs, in daily contact with a large number of inhaled aeropollutants and pathogens, have an extensive immune defense provided by the closely linked functions of many cells. This immune defense maintains an optimal balance between localizing normal microflora in the bronchi and detecting and destroying pathogens. Cigarette smoke affects many pathways, disrupting the complex chain of processes that ensure adequate immune control.

The innate immune system has a wide arsenal of tools it uses to detect and eliminate pathogens. That the innate immune system is a serious tool for ensuring an immune response is demonstrated by the fact that invertebrates lacking adaptive immunity have been successful in controlling pathogens. There is also emerging evidence that innate immune cells, including macrophages, are capable of developing immunological memory after previous contact with a pathogen, a characteristic previously thought to be only a property of adaptive immunity [178]. Immunological memory is accompanied by epigenetic and metabolic reprogramming of the cells [179]. It involves enhancing the expression of pattern recognition receptors, which increases their sensitivity to certain pathogens, what has been termed “trained immunity” [180,181]. The LPS tolerance previously described can also be considered as an adaptive mechanism of cellular responses to an external stimulus, which results in a lower inflammatory response to repeated stimulation [178,182,183]. Respiratory viral infection with effector CD8 T cells has been shown to induce long-term immune memory in alveolar macrophages [184]. These cells may have sustained trained immunity against bacterial infection in the lungs through rapid induction of chemokines and neutrophilia. The current paradigm of trained immunity involves the formation of a prolonged activation of the innate immune system following exposure to certain stimuli, which can lead to an enhanced immune response to a repeated stimulus [185]. The role of trained immunity has been demonstrated in atherosclerosis, as the resulting enhanced innate immune response is non-specific and can induce prolonged excessive inflammatory responses [186,187,188]. However, the involvement of these mechanisms in the pathogenesis of COPD remains to be elucidated. Viral infection occurring prior to bacterial exposure has been shown to result in greater host susceptibility to secondary bacterial infection due to the impaired immune response of macrophages and dendritic cells [189]. These findings are of interest given the role of viral infections in the initiation of COPD exacerbations.

Thus, the innate immune system plays an important role in maintaining respiratory immunological homeostasis, but may be significantly impaired by smoking and COPD and contribute significantly to the development and progression of the disease.

Some clinical characteristics of COPD heterogeneity, such as the frequency of exacerbations, the development of emphysema and sputum hyperproduction may be related to some impaired mechanisms of the innate immune system, which is a promising area for future research.

## Data Availability

Not applicable.
